# A novel NLRP3 inhibitor as a therapeutic agent against monosodium urate-induced gout

**DOI:** 10.3389/fimmu.2023.1307739

**Published:** 2024-02-02

**Authors:** Kihyoun Park, Injae Shin, Yoonseon Kim, Hyereen Kang, Soo-Jin Oh, Eunkyeong Jang, Taebo Sim, Jeehee Youn, Myung-Shik Lee

**Affiliations:** ^1^ Severance Biomedical Science Institute, Yonsei University College of Medicine, Seoul, Republic of Korea; ^2^ Graduate School of Medical Science, Brain Korea 21 Project, Yonsei University College of Medicine, Seoul, Republic of Korea; ^3^ Department of Biomedical Science, Hanyang University, Seoul, Republic of Korea; ^4^ Soonchunhyang Institute of Medi-bio Science and Division of Endocrinology, Department of Internal Medicine, Soonchunhyang University College of Medicine, Cheonan, Republic of Korea; ^5^ Department of Medicine, Hanyang University, Seoul, Republic of Korea

**Keywords:** inflammasome, NEK7, gout, monosodium urate, crystal

## Abstract

**Background:**

Since NEK7 is critical for NLRP3 inflammasome activation, NEK7 inhibitors could be employed as therapeutic agents against gout, a representative disease caused by NLRP3 inflammasome.

**Methods:**

We designed NEK7 inhibitors based on biochemical kinome profiling of 2,7-substituted thieno[3,2-d]pyrimidine derivatives (SLC3031~3035 and SLC3037). Inflammasome activation was assessed by ELISA of IL-1b and immunoblotting of IL-1b maturation after treatment of bone marrow-derived macrophages with LPS+monosodium urate (MSU). NLPR3 binding to NEK7 and oligomerization were examined using immunoprecipitation and Blue Native gel electrophoresis, respectively. In vivo effect was investigated by studying gross and histopathological changes of food pad tissue of MSU-injected mice, together with assays of maturation of IL-1b and ASC speck in the tissue.

**Results:**

SLC3037 inhibited inflammasome by MSU and other inflammasome activators through blockade of NLRP3 binding to NEK7 or oligomerization, and subsequent ASC oligomerization/phosphorylation. SLC3037 significantly reduced foot pad thickness and inflammation by MSU, which was superior to the effects of colchicine. SLC3037 significantly reduced content or maturation of IL-1b and ASC speck in the food pad. The number and height of intestinal villi were decreased by colchicine but not by SLC3037.

**Conclusion:**

SLC3037, a NLRP3 inhibitor blocking NEK7 binding to NLRP3, could be a novel agent against diseases associated with NLRP3 inflammasome activation such as gout, cardiovascular diseases, metabolic syndrome or neurodegenerative diseases.

## 1 Introduction

The inflammasome is a cytoplasmic multiprotein complex comprising a sensor protein, an adaptor protein ASC (apoptosis-associated speck-like protein containing a caspase-recruitment domain), and pro-caspase-1 (1). When the inflammasome complex is assembled through a homotypic interaction between PYD and CARD domains of sensor proteins and ASC, inactive pro-caspase-1 is auto-processed to active caspase-1, leading to maturation of pro-interleukin-1β (pro-IL-1β) or pro-IL-18 to their active forms (1, 2). There are several types of inflammasomes depending on the sensor proteins, and the NLRP3 inflammasome has been most extensively studied due to its potential contribution to the pathogenesis of numerous diseases such as cardiovascular, metabolic, neurodegenerative, and infectious diseases including COVID-19 infection (3–5).

Among diseases related to the NLRP3 inflammasome, gout is one of the first diseases pathogenically associated with the inflammasome (6). NLRP3 can be activated by diverse stimulators ranging from microbial products to host-derived danger signals or metabolites. Among diverse activators, monosodium urate (MSU) is a classical NLRP3 activator linked to the development of gout. MSU crystal belongs to lysosomotropic agents that can activate the inflammasome by acting on lysosomes after trapping into the lysosomal lumen (7, 8). Asymptomatic gout is managed by drugs inhibiting synthesis of uric acids such as allopurinol and febuxostat, or uricosuric agents. However, once gout attack due to MSU crystal deposition in tissues occurs, available drugs are only colchicine, corticosteroids, or non-steroidal anti-inflammatory drugs (9). Although colchicine has been employed as the drug of choice for gout attack for more than 200 years, its mechanism of action was recently elucidated: suppression of the microtubule-mediated NLRP3 inflammasome (6, 10). While clinically effective, colchicine has several adverse effects including severe gastrointestinal discomfort, diarrhea, bone marrow suppression, and hepatotoxicity (11). However, no drugs have been developed so far that can replace colchicine as the first-line drug against gout attack.

Assembly of the NLRP3 inflammasome during activation is regulated by several factors or events. Among them, NEK7, a member of the NIMA-related kinase (NEK) protein family, is critical in NLRP3 oligomerization as a downstream of K^+^ efflux (12, 13), which is another crucial event in most types of inflammasomes (14). After sensing a drop in intracellular K^+^ concentration, NLRP3 localization and structure are changed (15, 16). NEK7 localized to the centrosome bridges NLRP3 subunits recruited to microtubule-organizing center (MTOC) through dynein-mediated retrograde transport (17). In this process, NEK7 binds to the concave site of the LRR domain of NLRP3 and induces disassembly of the preassembled NLRP3 complex, exposure of the hidden PYD domain, and formation of the NLRP3 oligomer complex comprising ASC and pro-caspase-1 (15, 18). Thus, NEK7 can be a superb target for treatment of inflammasome-related diseases. Indeed, several drugs including MCC950, C1-27, IAA94, Rg3, and oridonin have been reported to suppress the inflammasome through NEK7 inhibition (19–21). In particular, MCC950 binds to the LRR domain of NLRP3 and stabilizes closed NLRP3 conformation inhibiting NEK7 binding (18). However, most of these agents were identified in systems unrelated to inflammasome and later found to have inhibitory activity on the inflammasome (18, 21–23).

In an attempt to develop novel inhibitors of the NLRP3 inflammasome acting on NEK7, we identified an NLRP3 inhibitor blocking NEK7 binding to NLRP3 and investigated its effect on an MSU-induced gout animal model, which mimics human gout arthritis pathogenically linked to the inflammasome.

## 2 Materials and methods

### 2.1 Chemistry

*General information*: Unless otherwise described, all commercial reagents and solvents were purchased from commercial suppliers and used without further purification. All reactions were performed under a N_2_ atmosphere in flame-dried glassware. The progress of reactions was monitored by using TLC with 0.25 mm E. Merck-precoated silica gel plates (60 F254), and a UV lamp, ninhydrin, or *p*-anisaldehyde stain for detection purposes. All solvents were purified by using standard techniques. Purification of reaction products was carried out by using silica gel column chromatography using Kieselgel 60 Art. 9385 (230–400 mesh). The purity of all compounds was ≥95%, and mass spectra and the purities of all compounds were analyzed using the Waters LCMS system (Waters 2998 Photodiode Array Detector, Waters 3100 Mass Detector, Waters SFO System Fluidics Organizer, Water 2545 Binary Gradient Module, Waters Reagent Manager, Waters 2767 Sample Manager) using the SunFire™ C18 column (4.6 × 50 mm, 5 μm particle size): solvent gradient = 30% B at 0.00 min, 30% B at 1.00 min, 100% B at 7.00 min, 100% B at 8.00 min, 30% B at 8.01 min, 30% B at 10.00 min. Solvent A consisted of 0.1% HCOOH in H_2_O and solvent B 0.1% HCOOH in MeOH, with a flow rate of 0.8 ml/min. The % purity of all compounds was analyzed at wavelengths of 254 nm, 275 nm, and 300 nm. The ^1^H and ^13^C NMR spectra were obtained using a Bruker 400 MHz FT-NMR (400 MHz for ^1^H and 100 MHz for ^13^C) spectrometer. Standard abbreviations are used for denoting the signal multiplicities.

**Figure 1:**
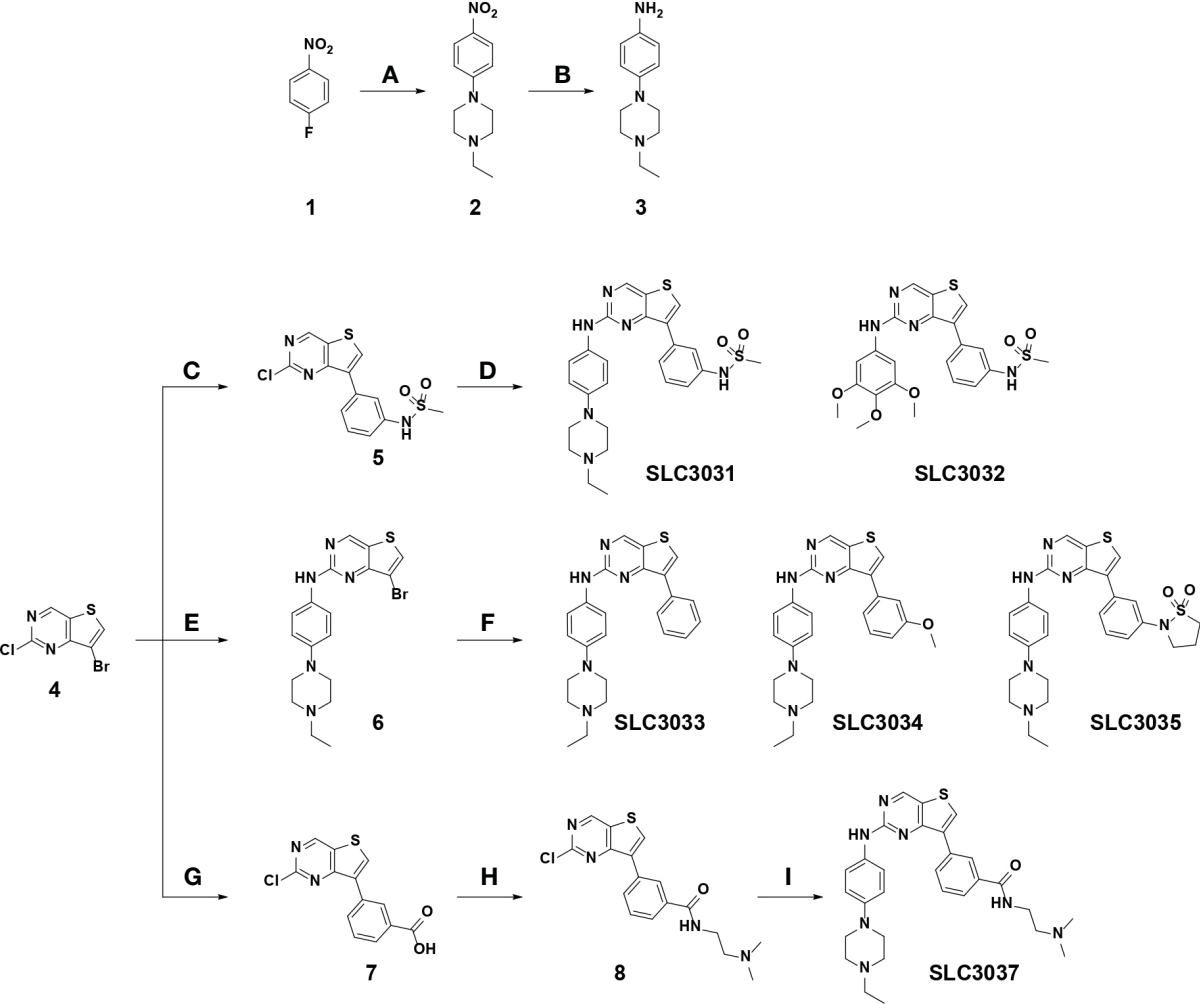
Synthesis of the NEK7 inhibitor. SLC3031~3035 and SLC3037 were synthesized as described in “Materials and methods”. Reagent and condition for these reactions are as follows: **(A)** K_2_CO_3_, 1-ethylpiperazine, DMSO, 100°C, 3 h, 85%–89%; **(B)** Pd/C, H_2_, EtOAc, RT, 12 h, 91%–95%; **(C)** (3-(N-methylsulfonamido)phenyl)boronic acid, 2 N Na_2_CO_3_, Pd(PPh_3_)_2_Cl_2_, t-BuXPhos, 1,4-dioxane, 100°C, 3 h, 73%; **(D)** various amines, K_2_CO_3_, Pd_2_dba_3_, XPhos, 2-BuOH, 100°C, 1 h, 76%–78%; **(E)** 4-(4-ethylpiperazin-1-yl)aniline, TFA, 2-BuOH, 120°C, 24 h, 73%; **(F)** various boronic acid, 2 N Na_2_CO_3_, Pd(PPh_3_)_2_Cl_2_, t-BuXPhos, 1,4-dioxane, 100°C, 3 h, 19-71% **(G)** 3-boronobenzoic acid, 2 N Na_2_CO_3_, Pd(PPh_3_)_2_Cl_2_, t-BuXPhos, 1,4-dioxane, 100°C, 3 h; **(H)** N,N-dimethylethylenediamine, HATU, DIPEA, DMF, RT, 1 h, 54% over 2 steps; **(I)** 4-(4-ethylpiperazin-1-yl)aniline, K_2_CO_3_, Pd_2_dba3, XPhos, 2-BuOH, 100°C, 1 h, 69%. (RT, room temperature).

*1-Ethyl-4-(4-nitrophenyl)piperazine* (*2 in* **Figure 1**): To a solution of 1 in **Figure 1** (1.0 g, 7.09 mmol) in DMSO (7.1 ml), K_2_CO_3_ (1.96 g, 14.18 mmol) and 1-ethylpiperazine (1.80 ml, 14.18 mmol) were added. The reaction mixture was then stirred for 1 h at room temperature, quenched with water, and diluted with EtOAc. The organic layer was washed with brine, dried over MgSO_4_, filtered, and concentrated. The residue was purified by flash column chromatography on silica gel (0% to 10% MeOH/DCM) to afford 2 (1.5 g, 89% yield) as a yellow solid. ^1^H NMR (400 MHz, DMSO-*d*_6_) δ ^1^H NMR (400 MHz, DMSO-*d*_6_) δ 8.04-8.00 (m, 2H), 7.01-6.97 (m, 2H), 3.42-3.40 (m, 4H), 2.45-2.43 (m, 4H), 2.33 (q, *J* = 7.19 Hz, 2H), 1.01 (t, *J* = 7.15 Hz, 3H); ^13^C NMR (100 MHz, DMSO-*d*_6_) δ 154.8, 136.8, 125.7, 112.5, 52.0, 51.5, 46.3, 12.0. LRMS (ESI) *m/z* 236 [M + H]^+^.

*4-(4-Ethylpiperazin-1-yl)aniline* (*3 in* **Figure 1** *)*: To a solution of 2 of **Figure 1** (1.0 g, 4.26 mmol) in EtOAc (14 ml) under H_2_, Pd/C (100 mg) was added. The reaction mixture was then stirred for 12 h at room temperature, filtered through a pad of Celite, washed with EtOAc, and concentrated. The resulting residue (794 mg, 91% yield) was used for the next step without further purification. ^1^H NMR (400 MHz, DMSO-*d*_6_) δ 6.70-6.66 (m, 2H), 6.53-6.49 (m, 2H), 4.49 (bs, 2H), 2.93-2.90 (m, 4H), 2.49-2.47 (m, 5H), 2.37 (q, *J* = 7.13 Hz, 2H), 1.03 (t, *J* = 7.21 Hz, 3H); ^13^C NMR (100 MHz, DMSO-*d*_6_) δ 142.4, 141.8, 117.7, 114.7, 52.5, 51.5, 50.2, 11.8. LRMS (ESI) *m/z* 206 [M + H]^+^.

*1-Ethyl-4-(6-methoxy-5-nitropyridin-2-yl)piperazine* (*5 in* **Figure 1**): The synthesis of 5 was described in our previous report (24).

*N-(3-(2-((4-(4-Ethylpiperazin-1-yl)phenyl)amino)thieno[3,2-d]pyrimidin-7-yl)phenyl)methanesulfonamide (SLC3031)*: The synthesis of SLC3031 was described in our previous report (24).

*N-(3-(2-((3,4,5-Trimethoxyphenyl)amino)thieno[3,2-d]pyrimidin-7-yl)phenyl)methanesulfonamide (SLC3032)*: The synthesis of SLC3032 was described in our previous report (24).

*7-Bromo-N-(4-(4-ethylpiperazin-1-yl)phenyl)thieno[3,2-d]pyrimidin-2-amine* (*6 in* **Figure 1**): The synthesis of 6 was described in our previous report (24).

*N-(4-(4-Ethylpiperazin-1-yl)phenyl)-7-phenylthieno[3,2-d]pyrimidin-2-amine (SLC3033)*: The synthesis of SLC3033 was described in our previous report (24).

*N-(4-(4-Ethylpiperazin-1-yl)phenyl)-7-(3-methoxyphenyl)thieno[3,2-d]pyrimidin-2-amine (SLC3034)*: The synthesis of SLC3034 was described in our previous report (24).

*2-(3-(2-((4-(4-Ethylpiperazin-1-yl)phenyl)amino)thieno[3,2-d]pyrimidin-7-yl)phenyl)isothiazolidine 1,1-dioxide (SLC3035)*: The synthesis of SLC3035 was described in our previous report (24).

*3-(2-Chlorothieno[3,2-d]pyrimidin-7-yl)-N-(2-(dimethylamino)ethyl)benzamide* (*8 in* **Figure 1**): To a solution of 4 in **Figure 1** (1 g, 4.02 mmol) in 1,4-dioxane (27 mL), 2 N Na_2_CO_3_ (12 mL, 12.05 mmol) and 3-boronobenzoic acid (730 mg, 4.42 mmol) were added. After flowing nitrogen over the solution for 10 min, Pd(PPh_3_)_2_Cl_2_ (140 mg, 0.20 mmol) and *t*-BuXphos (85 mg, 0.20 mmol) were added at room temperature. The reaction mixture was stirred at 100°C for 3 h and filtered through a Celite pad and concentrated. Dichloromethane (5 ml) and diethyl ether (45 ml) were added to the residue, stirred at room temperature for 1 h, and filtered. The resulting solid was washed with diethyl ether and dried at 50°C. To a solution of the resulting residue (600 mg) in DMF (10 ml) was added *N*,*N*-dimethylethylenediamine (0.45 ml, 4.14 mmol), HATU (1.6 g, 4.14 mmol), and DIPEA (1.1 ml, 6.21 mmol). The reaction mixture was then stirred for 1 h at room temperature, quenched with water, and diluted with EtOAc. The organic layer was washed with brine, dried over MgSO_4_, filtered, and concentrated. The resulting residue was purified by flash column chromatography on silica gel (0% to 10% MeOH/DCM) to afford 8 (781 mg, 54% yield over two steps) as a gray solid. ^1^H NMR (400 MHz, DMSO-*d*_6_) δ 9.58 (s, 1H), 8.88 (s, 1H), 8.73 (t, *J* = 5.44 Hz, 1H), 8.42 (t, *J* = 1.59 Hz, 1H), 8.18 (td, *J* = 1.21, 7.98 Hz, 1H), 7.92 (td, *J* = 1.28, 7.95 Hz, 1H), 7.67 (t, *J* = 7.76 Hz, 1H), 3.66 (q, *J* = 5.91 Hz, 2H), 3.30 (t, *J* = 6.05 Hz, 2H), 2.87 (s, 6H), 2.60 (bs, 1H); ^13^C NMR (100 MHz, DMSO-*d*_6_) δ 166.7, 159.3, 156.4, 155.8, 138.2, 134.3, 133.4, 132.5, 131.2, 130.9, 128.6, 127.0, 126.8, 56.1, 42.6, 39.4, 34.7. LRMS (ESI) *m/z* 361 [M + H]^+^.

*N-(2-(Dimethylamino)ethyl)-3-(2-((4-(4-ethylpiperazin-1-yl)phenyl)amino)thieno[3,2-d]pyrimidin-7-yl)benzamide (SLC3037)*: To a solution of 8 in **Figure 1** (300 mg, 0.83 mmol) in 2-butanol (5.5 ml), **3** (170 mg, 0.83 mmol), K_2_CO_3_ (572 mg, 4.15 mmol), Pd_2_(dba)_3_ (76 mg, 0.08 mmol), and XPhos (40 mg, 0.08 mmol) were added at room temperature. The reaction mixture was then stirred for 1 h at 100°C, cooled to room temperature, filtered, and concentrated. The resulting residue was purified by flash column chromatography on silica gel (0% to 20% MeOH/THF) to afford SLC3037 (302 mg, 69% yield) as a yellow solid. ^1^H NMR (400 MHz, DMSO-*d*_6_) δ 9.41 (s, 1H), 9.17 (s, 1H), 8.55 (s, 1H), 8.49 (s, 1H), 8.38 (t, *J* = 5.56 Hz, 1H), 8.20 (d, *J* = 7.82 Hz, 1H), 7.85 (d, *J* = 7.82 Hz, 1H), 7.73-7.71 (m, *J* = 9.05 Hz, 2H), 7.60 (t, *J* = 7.76 Hz, 1H), 6.87-6.85 (m, *J* = 9.05 Hz, 2H), 3.42 (q, *J* = 6.52 Hz, 2H), 3.08-3.05 (m, 4H), 2.52-2.50 (m, 4H), 2.44 (t, *J* = 6.97 Hz, 2H), 2.37 (q, *J* = 7.17 Hz, 2H), 1.03 (t, *J* = 7.15 Hz, 3H); ^13^C NMR (100 MHz, DMSO-*d*_6_) δ 166.2, 158.3, 157.9, 153.7, 146.0, 135.0, 134.1, 133.5, 132.7, 130.3, 128.2, 126.8, 126.2, 122.0, 119.9, 115.7, 58.0, 52.3, 51.5, 49.0, 45.1, 37.4, 11.8. LRMS (ESI) *m/z* 530 [M + H]^+^. HRMS (ESI) *m/z* calculated for C_29_H_36_N_7_OS^+^ [M + H]^+^: 530.2697. Found: 530.2691.

### 2.2 Biochemical *in vitro* kinase assay

Biochemical assay of the SLC3037 effect on NEK7 protein kinase activity was performed at Reaction Biology Corp. SLC3037 was tested in a 10-dose IC_50_ mode with threefold serial dilution starting at 10 µμ in the presence of 10 µμ of ATP.

### 2.3 Cell culture and drug treatment

Bone marrow-derived macrophages (BMDMs) were cultured in DMEM (Welgene, LM 001-05) containing 10% FBS, 100 U/ml penicillin, and 100 μg/ml streptomycin. For drug treatment, the following concentrations were used: MSU (500 μg/ml; Sigma, U2875), LPS (100 ng/ml; Sigma, L3024), SLC3037 (5 μM), LLOMe (400 nM; Sigma, L7393), nigericin (10 μM; Sigma, N7143), ATP (5 mM; Roche, 10127531001), and palmitic acid (PA) (300 μM; Sigma, P9767). PA stock solution (50 mM) was prepared by dissolving in 70% ethanol and heating at 55°C. Working PA solution was made by diluting PA stock solution in 2% fatty acid-free BSA-DMEM. For NLRP3 activation, BMDMs were treated with MSU, LLOMe, ATP, and nigericin PA for 5 h, 45 min, 45 min, 45 min and 16 h, respectively, after pretreatment with 100 ng/ml LPS for 4 (MSU) or 3 h (all other activators). For activation of AIM2 and NLRC4 inflammasomes, BMDMs pretreated with 100 ng/ml LPS for 3 h were transfected with 1 μg/ml poly(dA:dT) (Sigma, P0883) or 250 ng/ml flagellin (InvivoGen, tlrl-stfla) using Lipofectamine 2000 (Invitrogen) for 3 h. NEK7 inhibitors were added 1 h before addition of inflammasome activators or transfection, viz., after 2~3 h of incubation with LPS.

### 2.4 Antibodies and immunoblot analysis

Cells or tissues were solubilized in a lysis buffer containing protease inhibitors. The protein concentration was determined using the Bradford method. Samples (10~30 μg) were separated on 4%~12% Bis–Tris gel (NUPAGE, Invitrogen) and transferred to nitrocellulose membranes for immunoblot analysis using the ECL method (Dongin LS). To detect cleaved caspase-1 in cells treated *in vitro*, culture supernatant was precipitated with cold (−20°C) acetone, followed by resuspension in a sample buffer and heating at 100°C for 5 min before electrophoretic separation according to a previous protocol (25), since the intensity of the caspase-1 band in the BMDM extract was very faint. Antibodies against the following proteins were used for immunoblot analysis: IL-1β (R&D systems, AF-401-NA, 1: 1,000), caspase-1 p20 (Millipore, ABE1971, 1: 1,000), ASC (AdipoGen, AL177, 1: 1,000), phospho-ASC (ECM Biosciences, AP5631, 1: 1,000), NLRP3 (AdipoGen, AG-20B-0014, 1:1,000), NEK7 (Abcam, ab133514, 1:1,000), HSP 90 (Santa Cruz, sc13119, 1: 1,000), and β-actin (Santa Cruz, sc47778, 1: 1,000). Densitometry of the protein bands was performed using ImageJ software.

### 2.5 Immunoprecipitation

After lysis of cells in an ice-cold lysis buffer (400 mM NaCl, 25 mM Tris–HCl, pH 7.4, 1 mM EDTA, and 1% Triton X-100) containing protease and phosphatase inhibitors, lysates were centrifuged at 12,000*g* for 10 min in microfuge tubes and supernatant was incubated with anti-NEK7 antibody (Abcam, 1:1,000) or control IgG in a binding buffer (200 mM NaCl, 25 mM Tris–HCl, pH 7.4, 1 mM EDTA) with constant rotation at 4°C for 1 h. After adding 50 μl of 50% of Protein G bead (Roche) to lysates and incubating with rotation at 4°C overnight, resins were washed with a binding buffer. After resuspending pellet in a sample buffer and heating at 100°C for 3 min, supernatant was collected by centrifugation at 12,000*g* for 30 s, followed by electrophoretic separation in a NUPAGE gradient gel. Immunoblot analysis was conducted by sequential incubation with anti-NEK7 or -NLRP3 antibody as the primary antibody and then with horseradish peroxidase-conjugated anti-rabbit IgG or -mouse IgG. Bands were visualized using an ECL kit.

### 2.6 Detection of ASC oligomerization

ASC oligomerization was studied according to a published protocol (26). Briefly, BMDMs were washed in ice-cold PBS and then lysed in NP-40 buffer (20 mM HEPES–KOH pH 7.5, 150 mM KCl, 1% NP-40, and protease inhibitors). Lysate was centrifuged at 2,000*g*, 4°C for 10 min. The pellet was washed and resuspended in PBS containing 2 mM disuccinimidyl suberate (DSS) for crosslinking, followed by incubation at room temperature for 30 min. The samples were then centrifuged at 2,000*g*, 4°C for 10 min. Precipitated pellets and soluble lysates were subjected to immunoblot analysis using the anti-ASC antibody.

### 2.7 Blue-native PAGE

Blue-native polyacrylamide gel electrophoresis (BN-PAGE) was performed using the Bis–Tris NativePAGE system (Invitrogen), according to the manufacturer’s instructions. Briefly, cells were collected and lysed in 1× NativePAGE Sample Buffer containing 1% digitonin and protease inhibitor, followed by centrifugation at 13,000 rpm, 4°C, for 20 min. 20 μl supernatant mixed with 1 μl of 5% G-250 Sample Additive was loaded on a NativePAGE 3%~12% Bis–Tris gel. Samples separated on gels were transferred to PVDF membranes (Millipore) using a transfer buffer, followed by immunoblot analysis using the anti-NLRP3 antibody.

### 2.8 Immunofluorescence study

Tissue samples were fixed in 10% buffered formalin and embedded in paraffin. Sections of 4-μm thickness were incubated with anti-ASC antibody (1:200) as the primary antibody. After staining with anti-rabbit secondary antibody conjugated to Alexa Fluor 488 (Invitrogen) for 1 h, fluorescence was visualized with an LSM980 confocal microscope (Zeiss).

### 2.9 ELISA

Contents of IL-1β and TNF-α in culture supernatants of BMDMs or tissue extract were determined using mouse ELISA kits (DY401, R&D Systems for IL-1β and DY410, R&D Systems for TNF-α), according to the manufacturer’s instruction.

### 2.10 Animals

C57BL/6 mice were purchased from Orient Bio. All animal experiments were performed using 9-week-old male C57BL/6 mice after acclimation in a specific pathogen-free condition for 1 week before experiment. To induce acute gout arthritis, mice were injected subcutaneously with 3 mg of MSU crystals into the plantar surface of the hindfoot pad. 50 mg MSU was suspended in 1 ml of PBS and sonicated for 3 min on ice. 30 min and 24 h after MSU injection, each group of mice was treated by intraperitoneal injection of SLC3037, an NEK7 inhibitor (25 mg/kg, dissolved in 10% DMSO), or oral administration of colchicine (1 mg/kg, dissolved in 0.04% ethanol). To reduce pain, anesthesia was performed using isoflurane during the experiment. Euthanasia was performed if the weight was reduced by 20%. Foot pad thickness and %swelling were determined using a digimatic caliper. Hind paws were removed from mice 48 h after MSU injection, fixed, and decalcified in 5.5% EDTA in phosphate-buffered formalin before embedding in paraffin.

All animal experiments were conducted in accordance with the Public Health Service Policy in Humane Care and Use of Laboratory Animals. Animal protocols were approved by the IACUCs of Hanyang University and the Department of Laboratory Animal Resources of Yonsei University College of Medicine, AAALAC-accredited units. Temperature of animal rooms was 22 ± 2°C, and humidity was 50 ± 10%. Foot tissues were homogenized in RIPA buffer (50 mM Tris–HCl, pH 7.4, 1% NP-40, 0.25% sodium deoxycholate, 150 mM NaCl, 1 mM EGTA) containing protease inhibitors, and the homogenates were centrifuged at 12,000*g* for 10 min. Supernatant was subjected to ELISA or immunoblot analysis. To minimize any potential bias, all male mice were randomly assigned to experimental groups or age-matched control groups. Samples were prepared, treated, processed, and analyzed in random order. All samples were collected and analyzed under the same condition.

### 2.11 Blood chemistry and hemogram

Analysis of blood chemistry was conducted using a Fuji Dri-Chem analyzer. Hemogram was obtained using heparinized blood and a Hamevet950 Blood Analyzer (Drew Scientific).

### 2.12 Statistical analysis

All values are expressed as the means ± SEM of ≥3 independent experiments performed in triplicate. Two-tailed Student’s *t*-test was employed to compare values between two groups. One-way ANOVA with Tukey’s test was used to compare values between multiple groups. Two-way repeated-measures ANOVA with Bonferroni’s *post-hoc* test was used to compare multiple repeated measurements between groups. Levene’s test was employed to confirm the homogeneity of variances between multiple groups. GraphPad Prism 6 software was employed for statistical analysis. *P* values < 0.05 were considered significant.

## 3 Results

### 3.1 Inhibition of inflammasome *in vitro* by a NLRP3/NEK7 inhibitor

We have reported that 2,7-substituted thieno[3,2-*d*]pyrimidine derivatives possess potent inhibitory activities against Flt3 and FAK (24). In the course of exploring inhibitory activities of 2,7-substituted thieno[3,2-*d*]pyrimidine derivatives against other kinases by biochemical kinome profiling, we have found that a 2,7-substituted thieno[3,2-*d*]pyrimidine derivative could inhibit NEK7 (data not shown), which led us to explore 2,7-substituted thieno[3,2-*d*]pyrimidine derivatives as potential NEK7 inhibitors. Based on structural features of substituents of the thieno[3,2-*d*]pyrimidine derivatives identified by us, six derivatives (SLC3031 ~ 3035 and SLC3037) were selected to investigate their inhibitory activities against the inflammasome. A key intermediate, 7-bromo-2-chlorothieno[3,2-*d*]pyrimidine (4 in **Figure 1**), was synthesized according to the experimental procedures described in our previous report (24). The aromatic amine (3 in **Figure 1**) was prepared by nucleophilic aromatic substitution reaction of 4-fluoronitrobenzene with 1-ethylpiperazine (a), followed by reduction of the nitro group utilizing catalytic hydrogenation (b). At C-7 position of thieno[3,2-*d*]pyrimidine, various substituted phenyl groups were installed through palladium-mediated Suzuki coupling reactions (c, f, and g). Various amine analogues were introduced through Buchwald amination coupling reactions (d and **i**) and acid-catalyzed amination reactions (e) at the C-2 position of thieno[3,2-*d*]pyrimidine for synthesis of SLC3031 ~ 3035 and SLC3037 (**Figure 1**).

**Figure 2:**
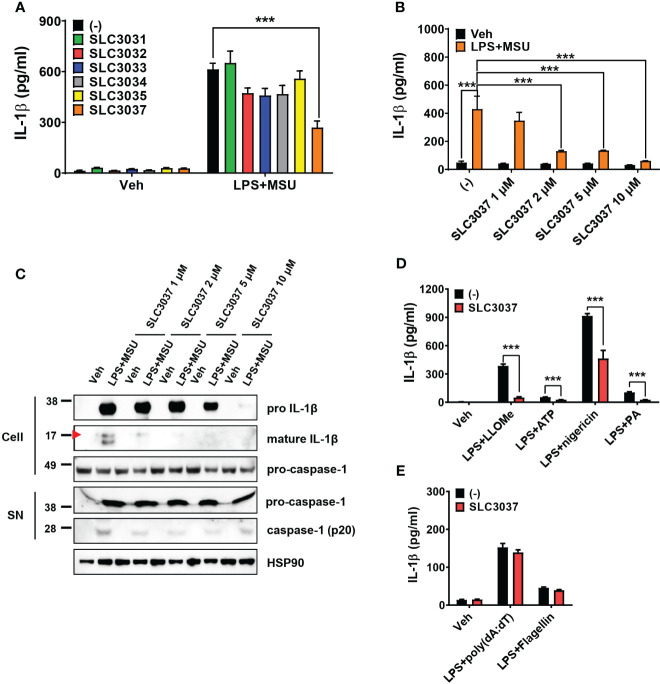
Inhibition of the inflammasome by SLC3037. **(A)** BMDMs were primed with 100 ng/ml LPS for 4 h and then treated with 500 μg/ml MSU for 5 h, in the presence or absence of NEK7 inhibitors. The IL-1β content in the culture supernatant was determined by ELISA (n = 3 each). **(B)** BMDMs were treated with LPS+MSU with varying concentrations of SLC3037. The IL-1β content in the culture supernatant was determined by ELISA (n = 4 each). **(C)** After treatment of BMDMs as in **(B)**, the cell extract or resuspended acetone precipitate of culture supernatant (SN) was subjected to immunoblot analysis using indicated antibodies. **(D)** BMDMs were primed with 100 ng/ml LPS for 3 h and then treated with LLOMe for 45 min, ATP for 45 min, nigericin for 45 min, or PA for 16 h in the presence or absence of SLC3037, and IL-1β content in the culture supernatant was determined by ELISA (n=4 each). **(E)** BMDMs pretreated with 100 ng/ml LPS for 3 h were transfected with poly(dA:dT) or flagellin for 3 h in the presence or absence of SLC3037, and the IL-1β content in the culture supernatant was determined by ELISA (n = 4 each). All data in this figure are the means ± SEM from more than three independent experiments. Red arrowheads indicate bands representing mature IL-1β. ***p < 0.001 by one-way ANOVA with Tukey’s test.

Using these compounds, we studied whether SLC3031~3035 and SLC3037 at 5μM concentration could inhibit IL-1β release from bone marrow-derived macrophages (BMDMs) in response to MSU, an effector of inflammasome activation in gout (6) in combination with LPS (LPS+MSU). ELISA of culture supernatant showed that SLC3037 significantly suppressed IL-1β release in response to LPS+MSU (613.56 ± 36.18 for (−):LPS+MSU versus 269.07 ± 38.74 pg/ml for SLC3037:LPS+MSU, p < 0.0001 by one-way ANOVA with Tukey’s test) (**Figure 2A**). Effects of SLC3031, SLC3032, SLC3033, SLC3034, and SLC3035 were marginal without statistical significance (**Figure 2A**). Thus, we employed SLC3037 for further studies. Biochemical *in vitro* kinase assay confirmed that SLC3037 inhibits kinase activity of NEK7 with an IC_50_ value of 2.85 μM (**Supplementary Figure 1**). When we studied the dose–response relationship, SLC3037 inhibited both IL-1β release and maturation of pro-IL-1β to IL-1β after LPS+MSU treatment in a dose-dependent manner between 1μM and 10μM concentrations (48.26 ± 10.45 for (−):Veh versus 429.68 ± 91.54 pg/ml for (−):LPS+MSU, p < 0.0001) (347.65 ± 58.86 pg/ml for 1 μM SLC3037:LPS+MSU, p > 0.1; 129.67 ± 5.73 pg/ml for 2 μM SLC3037:LPS+MSU, p < 0.0001; 134.29 ± 2.78 pg/ml for 5 μM SLC3037:LPS+MSU, p < 0.0001; 59.88 ± 1.38 pg/ml for 10 μM SLC3037:LPS+MSU, p < 0.0001 all versus (−):LPS+MSU by one-way ANOVA with Tukey’s test) (**Figure 2B**), suggesting that SLC3037 is an authentic inhibitor of inflammasome. Immunoblot analysis also demonstrated that SLC3037 inhibits maturation of pro-IL-1β to IL-1β in BMDMs treated with LPS+MSU (**Figure 2C**), validating the results of ELISA. Cleavage of pro-casapase-1 to active caspase-1 after LPS+MSU treatment was also inhibited by SLC3037 when acetone precipitate of culture supernatant was employed for immunoblot analysis (**Figure 2C**). Cleaved caspase-1 was not visible when the BMDM extract was employed. When inflammasome activators other than MSU were employed, IL-1β release in response to LPS + LLOMe, ATP, nigericin, or PA in combination with LPS was significantly reduced by SLC3037 (384.10 ± 20.48 for (−):LPS+LLOMe versus 50.05 ± 7.47 pg/ml for SLC3037:LPS+LLOMe, p < 0.0001; 54.31 ± 1.62 for (−):LPS+ATP versus 26.70 ± 0.94 pg/ml for SLC3037;LPS+ATP, p < 0.0001; 915.52 ± 23.31 for (−):LPS+nigericin versus 463.45 ± 85.48 pg/ml for SLC3037:LPS+nigericin, p < 0.01; 103.84 ± 7.55 for (−):LPS+PA versus 25.51 ± 2.68 pg/ml for SLC3037:LPS+PA, p < 0.0001 by two-tailed Student’s *t*-test) (**Figure 2D**), suggesting that SLC3037 could inhibit inflammasome activation by multiple activators. In contrast to the inhibition of IL-1β release by SLC3037, TNF-α release in response to MSU in combination with LPS was not significantly decreased by multiple concentrations of SLC3037, suggesting specific inhibition of inflammasome by SLC3037 (p > 0.1 by one-way ANOVA with Tukey’s test) (**Supplementary Figure 2A**). TNF-α release in response to LLOMe or ATP in combination LPS was also not significantly reduced by SLC3037 (p > 0.1 by two-tailed Student’s *t*-test), although TNF-α release in response to nigericin or PA in combination with LPS tended to be downregulated by SLC3037 (p < 0.01 by two-tailed Student’s *t*-test), which could be due to the effects of IL-1β on the secondary cytokine release (**Supplementary Figure 2B**). To corroborate the specific inhibition of the NRLP3 inflammasome by SLC3037, we studied whether SLC3037 affects IL-1β release in response to poly(dA:dT) and flagellin, activators of AIM2 and NLRC4 inflammasome, respectively (1, 13). IL-1β release after transfection of LPS-pretreated BMDMs with poly(dA:dT) or flagellin was not significantly inhibited by SLC3037 (p > 0.1 and p > 0.05, respectively, by two-tailed Student’s *t*-test) (**Figure 2E**), indicating that SLC3037 does not affect NEK7-independent inflammasome activation (13).

**Figure 3:**
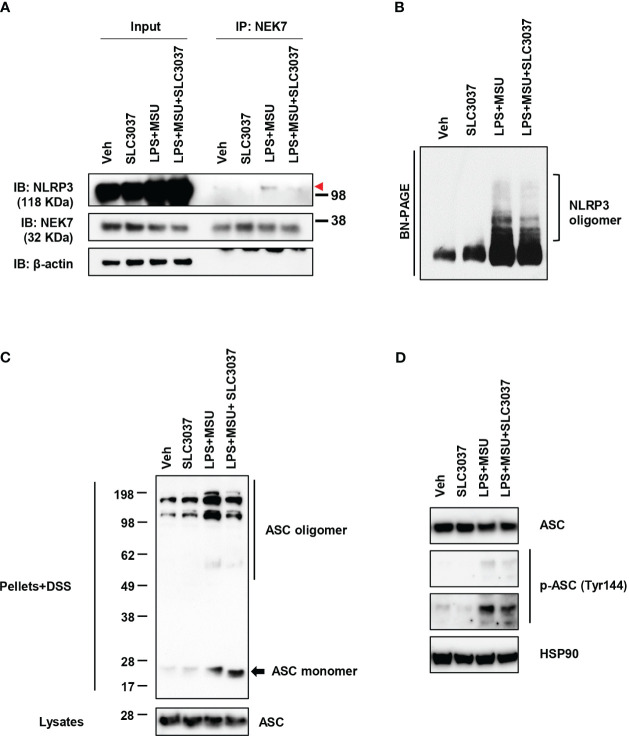
Inhibition of NLRP3 and ASC oligomerization by SLC3037. **(A)** BMDMs were treated with LPS+MSU in the presence or absence of SLC3037. The cell lysate was immunoprecipitated using the anti-NEK7 antibody, which was subjected to immunoblot analysis using the indicated antibodies as described in “Materials and methods” (red arrowhead, NLRP3 band) **(B)** After treating BMDMs as in **(A)**, the cell lysate was separated on a Blue native gel, which was subjected to immunoblot analysis using anti-NLRP3 antibody. **(C)** BMDMs were treated with LPS+MSU in the presence or absence of SLC3037. The cell extract was subjected to immunoblot analysis using anti-ASC antibody after DSS crosslinking. **(D)** Cells were treated as in **(C)**, and the cell extract was subjected to immunoblot analysis using anti-p-ASC (Tyr144) antibody.

We next studied whether SLC3037 could inhibit NEK7 binding to NLRP3, a critical process in inflammasome activation (27). Immunoprecipitation study showed apparently reduced NEK7 binding to NLRP3 by SLC3037 (**Figure 3A**), suggesting that SLC3037 inhibits inflammasome activation via inhibition of NEK7-NLRP3 interaction. We also studied NLRP3 oligomerization, which is a hallmark of inflammasome activation and relies on NLRP3 binding to NEK7 bridging NLRP3 oligomerization (27). Likely due to inhibition of NEK7 binding to NLRP3, NLRP3 oligomerization was apparently reduced by SLC3037 (**Figure 3B**), suggesting that SLC3037 inhibits the inflammasome through inhibition of NLRP3 binding to NEK7 and oligomerization. We next studied ASC oligomerization, which occurs after rearrangement of NLRP3 cages into active oligomers in association with NEK7 (28). Consistent with the effect of SLC3037 on the IL-1β release and NLRP3 binding to NEK7, SLC3037 reduced ASC oligomerization by LPS+MSU (**Figure 3C**), indicating that SLC3037 inhibits the inflammasome through suppression of ASC oligomerization. We also studied ASC phosphorylation, which is crucial for ASC oligomerization and inflammasome activation (29). Again, SLC3037 inhibited ASC phosphorylation by LPS+MSU (**Figure 3D**), supporting that SLC3037 inhibits the inflammasome through blockade of ASC phosphorylation and oligomerization.

### 3.2 Inhibition of MSU-induced gout by the NLRP3/NEK7 inhibitor

**Figure 4:**
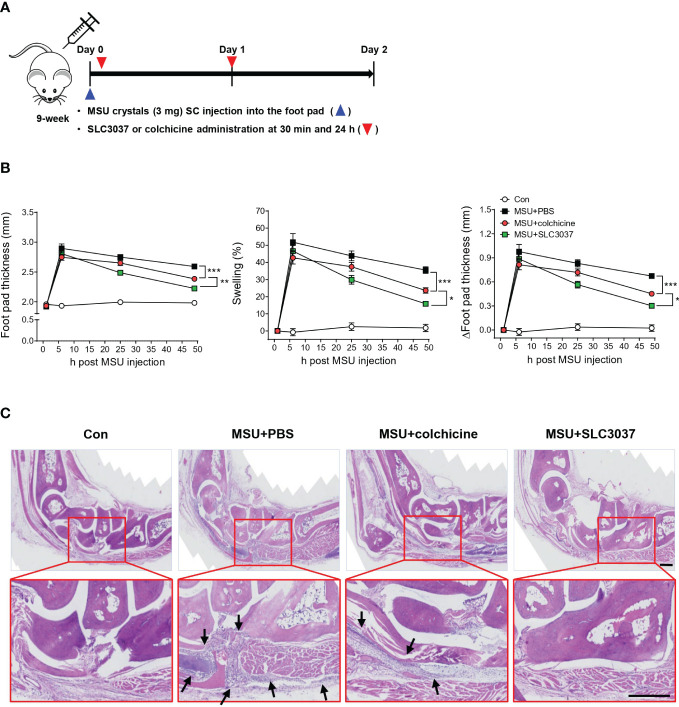
*In vivo* effect of SLC3037 on MSU-induced gout. **(A)** Schematic drawing of the time of MSU, SLC3037, or colchicine administration. (SC, subcutaneous) **(B)** MSU was subcutaneously injected into the foot pad of mice, and foot pad thickness, %swelling, and Δfoot pad thickness were determined using a digimatic caliper (n = 16 each). (Con, control) **(C)** H&E staining of foot pad sections from mice of **(B)** (arrows, foci of inflammation) (scale bar: 100 μm). All data in this figure are the means ± SEM from more than three independent experiments. Rectangles were magnified. *p < 0.05, **p < 0.01, ***p < 0.001 by two-way ANOVA with Bonferroni’s test.

Since it is well established that MSU crystal can elicit inflammasome-dependent gout in animal models (30), we examined whether SLC3037 could inhibit clinical manifestations of gout *in vivo*, using an MSU crystal-induced gout model (30). When MSU was injected subcutaneously into the foot pad of mice, swelling of the foot pad was well observed, as evidenced by increased total foot pad thickness, %swelling, and Δfoot pad thickness, likely due to *in vivo* inflammasome activation by MSU (**Figures 4A, B**). Foot pad swelling reached a peak 5 h after injection and waned after 24 h~48 h (**Figure 4B**). When SLC3037 was injected twice at 30 min and 24 h after MSU injection, foot pad swelling was significantly reduced as evidenced by decreased total foot pad thickness, %swelling, and Δfoot pad thickness compared with PBS-treated mice (foot pad thickness at 48 h: 2.59 ± 0.02 for MSU+PBS versus 2.22 ± 0.03 mm for MSU+SLC3037, p < 0.0001; %swelling at 48 h: 35.51 ± 1.90 for MSU+PBS versus 15.84 ± 1.06 for MSU+SLC3037, p < 0.0001; Δfoot pad thickness at 48 h: 0.67 ± 0.02 for MSU+PBS versus 0.30 ± 0.02 mm for MSU+SLC3037, p < 0.0001 by two-way ANOVA with Bonferroni’s test) (**Figure 4B**), suggesting suppression of MSU-induced tissue inflammation *in vivo* by SLC3037. We also examined the effect of colchicine dosed at 1 mg/kg, which has been used to treat MSU-induced gout in experimental animals (30). This dose of colchicine is equivalent to 0.081 mg/kg for human patients (31) and is higher than the dose for human patients with acute gout attack (9, 32). Although colchicine significantly reduced foot pad thickness, %swelling, and Δfoot pad thickness 48 h after MSU injection (foot pad thickness: 2.38 ± 0.03 mm for MSU+colchicine, p < 0.001 versus MSU+PBS; %swelling: 23.60 ± 1.54 for MSU+ colchicine, p < 0.001 versus MSU+PBS; Δfoot pad thickness: 0.45 ± 0.03 mm for MSU+colchicine, p < 0.001 versus MSU+PBS by two-way ANOVA with Bonferroni’s test), the decreases of total foot pad thickness, %swelling, and Δfoot pad thickness were significantly less than those in MSU-injected mice treated with SLC3037 (foot pad thickness: p < 0.01; %swelling: p < 0.05; Δfoot pad thickness: p < 0.05 by two-way ANOVA with Bonferroni’s test) (**Figure 4B**), suggesting a higher efficacy of SLC3037 compared with colchicine in treating acute gout attack.

When we studied histopathologic changes in hind paws 48 h after injection, MSU-treated mice exhibited marked inflammatory cell infiltration in subcutaneous soft tissue and joint space, which could explain tissue swelling (**Figure 4C**). Here, administration of SLC3037 dramatically reduced inflammatory cell infiltration in soft tissue of the foot pad. Colchicine also reduced the inflammatory cell infiltration albeit to an apparently lesser extent compared with SLC3037 (**Figure 4C**), consistent with the aforementioned clinical results demonstrating higher efficacy of SLC3037 compared with colchicine.

### 3.3 Inhibition of MSU-induced inflammasome activation *in vivo* by the NLRP3/NEK7 inhibitor

**Figure 5:**
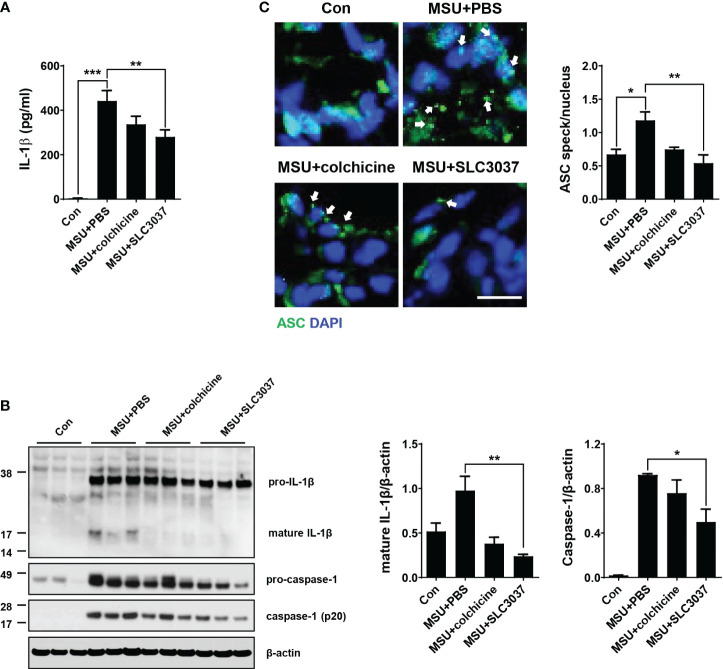
Inhibition of MSU-induced inflammasome by SLC3037 in vivo. **(A)** Two days after subcutaneous injection of MSU to the foot pad (i.e., one day after the last administration of colchicine or SLC3037), IL-1β content in tissue extract of the foot pad was determined by ELISA (n=10 each). **(B)** Solubilized tissue extract of **(A)** was subjected to immunoblot analysis using indicated antibodies (left). Densitometric analysis of immunoblot bands was conducted (right) (n=3 each). **(C)** Paraffin sections of foot pad tissue from mice of (A) were subjected to immunofluorescence using anti-ASC antibody, and the number of ASC specks (arrows) was counted (n=3 each) (right). Representative immunofluorescence images are shown (left panel). (scale bar: 10 μm) All data in this figure are the means ± SEM from more than three independent experiments. *p < 0.05, **p < 0.01, ***p < 0.001 by one-way ANOVA with Tukey’s test.

We next studied whether improved clinical manifestation of gout and inflammation by the NEK7 inhibitor are due to inhibition of MSU-induced inflammasome *in vivo*. When the IL-1β content in tissue extract of the foot pad from MSU-treated mice was determined using ELISA, significant IL-1β was detected (**Figure 5A**), indicating inflammasome activation by MSU. SLC3037 administration significantly suppressed IL-1β content in foot pad tissue of mice treated with MSU (442.31 ± 46.46 for MSU+PBS versus 280.30 ± 31.88 pg/ml for MSU+SLC3037, p < 0.01 by one-way ANOVA with Tukey’s test) (**Figure 5A**). In contrast, the decrease of IL-1β content in foot pad tissue of MSU-treated mice by colchicine was statistically insignificant (**Figure 5A**). Immunoblot of foot pad tissue extract demonstrated maturation of pro-IL-1β to IL-1β and caspase-1 cleavage by MSU injection (**Figure 5B**), consistent with inflammasome activation by MSU. SLC3037 administration significantly suppressed maturation of pro-IL-1β to IL-1β and caspase-1 cleavage in tissue extract of the foot pad by MSU as determined by densitometric analysis (IL-1β: 0.98 ± 0.16 for MSU+PBS versus 0.24 ± 0.02 for MSU+SLC3037, p < 0.01; caspase-1: 0.92 ± 0.01 for MSU+PBS versus 0.50 ± 0.12 for MSU+SLC3037, p < 0.05 by one-way ANOVA with Tukey’s test), indicating inhibition of inflammasome activation (**Figure 5B**). Colchicine also decreased pro-IL-1β maturation to IL-1β and caspase-1 cleavage by MSU; however, the decreases were statistically insignificant (**Figure 5B**), suggesting a stronger suppressive effect of SLC3037 on the inflammasome compared with colchicine. In contrast to the inhibition of IL-1β release by SLC3037 *in vivo*, the content of TNF-α in the foot pad lysate of mice injected with MSU was not significantly reduced by SLC3037 administration, suggesting specific inhibition of the inflammasome by SLC3037 *in vivo* (p > 0.05 by one-way ANOVA) (**Supplementary Figure 2C**).

Additionally, we studied ASC speck, a hallmark of tissue inflammasome activation (29). In the foot pad of MSU-injected mice, ASC speck was well observed by confocal microscopy after immunostaining with anti-ASC antibody (**Figure 5C**), indicating inflammation activation. SLC3037 significantly reduced the number of ASC speck in foot pad tissue of MSU-treated mice (1.18 ± 0.13 in MSU+PBS versus 0.54 ± 0.12 in MSU+SLC3037, p < 0.01 by one-way ANOVA with Tukey’s test) (**Figure 5C**), showing suppression of MSU-induced inflammasome by SLC3037. Colchicine also decreased the number of ASC speck in the foot pad of MSU-treated mice; however, the decrease was statistically insignificant (**Figure 5C**), consistent with a stronger suppressive effect of SLC3037 on the MSU-induced inflammasome compared with colchicine.

**Figure 6:**
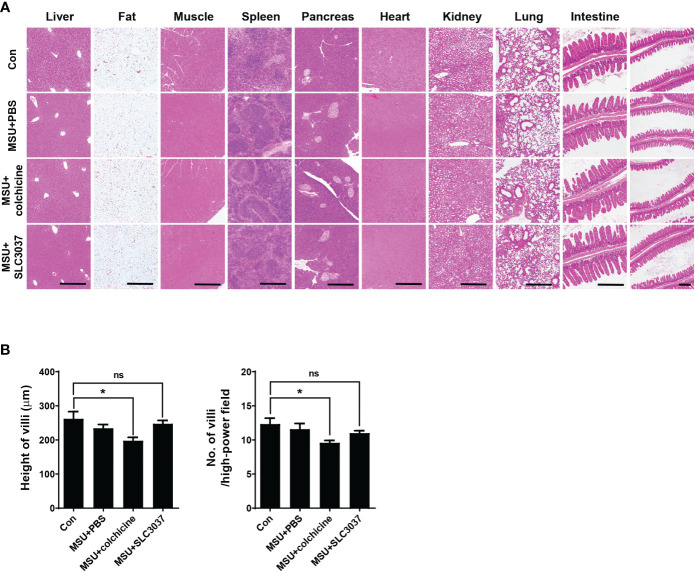
Biopsy of major organs after *in vivo* administration of SLC3037. **(A)** Major organs were obtained from C57BL/6 mice injected with MSU and then treated with SLC3037 or colchicine, which were subjected to histological analysis after H&E staining. (scale bar, 500 μm) **(B)** Height and number of intestinal villi were calculated from the pictures of **(A)** labelled as “intestine” (n = 4 each). All data in this figure are the means ± SEM from more than three independent experiments. *p < 0.05, by one-way ANOVA with Tukey’s test. ns, not significant.

To study the side effects of SLC3037, we investigated possible changes of blood cell number and chemistry. Administration of SLC3037 or colchicine to MSU-injected mice twice in 2 days did not cause specific abnormality in hemogram or blood chemistry (**Tables 1A, B**). We also conducted major organ biopsy to study the possible occurrence of histopathological abnormality by SLC3037. H&E staining of tissue sections did not reveal detectable abnormalities in the major organs including the liver, adipose tissue, skeletal muscle, spleen, pancreas, heart, kidney, and lung of mice treated with SLC3037 or colchicine (**Figure 6A**). However, in colchicine-treated mice, decreased height and number of intestinal villi were observed (height: 262.00 ± 21.10 for Con versus 197.59 ± 9.98 μm for MSU+colchicine, p < 0.05; number: 12.33 ± 0.85 for Con versus 9.58 ± 0.34/high-power field for MSU+colchicine, p < 0.05 by one-way ANOVA with Tukey’s test) (**Figures 6A, B**), which could be due to impaired mitosis of highly proliferative intestinal epithelial cells, consistent with a previous paper (33). In contrast, such abnormalities in the intestinal villi were not observed in SLC3037-treated mice (height: 247.48 ± 9.80 μm for MSU+SLC3037, p > 0.1 versus Con; number: 11.00 ± 0.36/high-power field for MSU+SLC3037, p > 0.1 versus Con by one-way ANOVA with Tukey’s test) (**Figures 6A, B**), suggesting no severe adverse effects of SLC3037 on the intestinal epithelium in contrast to colchicine. These results suggest that SLC3037 has not only a higher efficacy but also less adverse effect compared with colchicine in treating an animal model of gout.

**Table 1:** Laboratory profile of mice treated with SLC3037 or colchicine.

A							
		Unit	Ref. ranges	Con	MSU+PBS	MSU+colchicine	MSU+SLC3037
Leukocytes	WBC	K/μl	1.8-10.7	5.14±1.73	5.69±2.22	3.82±1.39	4.00±0.97
	NEU	K/μl	0.1-2.4	0.45±0.21	0.63±0.21	0.48±0.35	0.79±0.64
	LYM	K/μl	0.9-9.3	4.26±1.60	4.71±2.13	3.07±1.31	2.94±0.88
	MONO	K/μl	0.0-0.4	0.34±0.15	0.31±0.08	0.19±0.12	0.23±0.08
	EOS	K/μl	0.0-0.2	0.07±0.05	0.03±0.01	0.04±0.02	0.03±0.01
	BASO	K/μl	0.0-0.2	0.02±0.02	0.01±0.01	0.01±0.01	0.01±0.01
Erythrocytes	RBC	M/μl	6.36-9.42	9.94±0.59	10.14±0.28	9.16±1.85	9.89±0.44
	HGB	g/dL	11.0-15.1	12.96±1.38	13.25±0.85	12.28±2.78	13.18±1.55
	HCT	%	35.1-45.4	56.21±1.93	57.38±1.70	51.61±10.36	54.90±1.94
Thrombocytes	PLT	K/μl	592-2972	1120.00±157.16	1239.88±126.54	1088.50±222.77	1154.00±202.90

B							
				Con	MSU+PBS	MSU+colchicine	MSU+SLC3037
AST (U/l)				115.00±11.18	132.00±23.87	116.00±14.32	112.00±21.10
ALT (U/l)				58.00±2.74	60.00±3.54	57.00±2.74	59.00±4.18
TG (mg/dl)				79.00±36.30	38.00±16.43	41.00±17.82	25.00±16.96
TCHO (mg/dl)				80.00±3.54	86.00±10.25	83.00±6.71	90.00±3.54
ALP (U/l)				564.00±41.14	548.00±51.79	611.00±112.10	477.00±55.07
ALB (g/dl)				1.00±0.00	1.20±0.27	1.00±0.00	1.00±0.00
DBIL (mg/dl)				<0.05	<0.05	<0.05	<0.05
GGT (U/l)				<0.5	<0.5	<0.5	<0.5
LDH (U/l)				639.00±61.07	626.00±191.36	502.00±98.53	478.00±165.82
CRE (mg/dl)				1.04±0.07	1.08±0.06	1.08±0.07	1.03±0.08
CPK (U/l)				1859.00±321.62	2349.00±761.94	1509.00±327.14	1716.00±731.64
CA (mg/dl)				13.20±0.27	13.10±0.22	13.20±0.07	13.30±0.27
BUN (U/l)				20.00±2.03	18.70±3.17	17.40±1.92	17.60±1.78

No abnormality in hemogram **(A)** or blood chemistry **(B)** was detected in C57BL/6 injected with MSU and then treated with SLC3037 or colchicine. Con, control.

## 4 Discussion

In our attempt to develop a novel inflammasome inhibitor blocking NEK7 action, we identified SLC3037, which could inhibit inflammasome activation by MSU, an effector of inflammasome activation associated with gout. As an inhibitor or NEK7, SLC3037 would inhibit the activating step of the inflammasome rather than the priming step, since the pro-caspase-1 level in BMDMs and the pro-IL-1β level in food pad tissue were not affected by SLC3037. The marked decrease of pro-IL-1β in BMDMs treated with 10 μM SLC3037 *in vitro* but not in those treated with 1~5 μM SLC3037 might be due to the leakage of pro-IL-1β to the culture supernatant associated with cell death such as pyroptosis by a high concentration of SLC3037 rather than inhibition of pro-IL-1β expression since the pro-IL-1β level was not notably reduced by 1~5 μM SLC3037 despite significantly reduced release of mature IL-1β. Reduced expression of pro-caspase-1 in food pad tissue of SLC3037-treated mice is not likely to be a direct effect of SLC3037 since pro-caspase-1 expression was not reduced by SLC3037 treatment *in vitro* and might be due to an indirect effect related to a cell-extrinsic mechanism. These results suggest that SLC3037 inhibits the activating step rather than the priming step of inflammasome activation.

Contribution of NEK7 inhibition in SLC3037-mediated suppression of the inflammasome *in vitro* was most clearly shown by the inhibition of NEK7 binding to NLRP3 and NLRP3 oligomerization by SLC3037, as NEK7 binding to the concave LRR domain of NLRP3 and subsequent formation of a large NEK7–NLRP3 oligomer complex are critical steps in inflammasome activation (15, 18). NEK7 inhibitors such as SLC3037 might interfere with NEK7 binding to LRR of NLRP3 and inhibit NEK7 action to open the NLRP3 cage, which is critical for the rearrangement of NLRP3 into active oligomer (28). However, as SLC3037 was originally derived from known FAK inhibitors, the possibility that FAK inhibition had a synergistic effect on inflammasome inhibition cannot be totally eliminated (34). In addition to *in vitro* effects, SLC3037 also inhibited indices of clinical gout and *in vivo* inflammasome by MSU administration, suggesting the potential of SLC3037 as a new drug against gout attack. The effect of SLC3037 was more pronounced than that of colchicine, which has been the drug of choice against gout attack for centuries but can cause several untoward effects in patients. In addition, adverse effects on intestinal villi such as decreased height and number, which was observed after colchicine administration, was absent in mice treated with SLC3037.

Although we identified SLC3037 as an inhibitor of kinase activity of NEK7, catalytic activity of NEK7 has been reported to be dispensable for NEK7 action in inflammasome activation (13). Inhibition of kinase activity of NEK7 might cause untoward effects unrelated to inflammasome inhibition, since NEK7 is an important kinase regulating mitotic spindle assembly and mitosis (35). Likely due to its limited cellular content, NEK7-mediated inflammasome activation and mitosis are mutually exclusive events (36), and regulation of inflammasome activation by NEK7 occurs only in the interphase of the cell cycle. Thus, cells with the inhibited inflammasome by SLC3037 are likely myeloid cells in the interphase. In mice treated with SLC3037, cells other than macrophages such as lymphoid or epithelial cells in mitosis might be affected by SLC3037. However, likely due to the short administration period, systemic side effects were not manifest as evidenced by no changes in hemogram, blood chemistry, or biopsy of major organs including the small intestine after *in vivo* administration of SLC3037 twice in 2 days. These data might have clinical relevance since small intestinal epithelial cells have the highest turnover rate in the whole body with renewal in less than 2 days in rodents (33), suggesting that SLC3037 administration twice in 2 days would not impair proliferation or mitosis of even the most highly proliferating cells in the body. Thus, the probability of significant systemic toxicity related to the inhibition of mitosis might be insignificant in patients with acute gout attack treated with SLC3037 for a short period. In contrast, the number and height of intestinal villi were significantly reduced by colchicine at the 1-mg/kg dose, which is consistent with previous results reporting gastrointestinal mucosal injury in mice treated with 0.5 mg/kg colchicine (37, 38). Adverse effects of colchicine on intestinal mucosal cells could be attributable to effects of colchicine binding to microtubule and inhibiting mitosis of intestinal epithelial cells (39).

In conclusion, we identified a novel NLRP3 inhibitor that has significant suppressive effects on the inflammasome by several NLRP3 activators *in vitro* including MSU. We also observed marked suppression of clinical manifestations of gout together with amelioration of the inflammasome *in vivo*. Our NLRP3 inhibitor appears to be more efficacious in the treatment of MSU-induced gout of experimental animals than colchicine and was devoid of adverse effects on intestinal mucosa, which was observed after treatment with colchicine. Since kinase activity of NEK7 is dispensable for NRLP3 activation and we did not prove direct binding of SLC3037 to NEK7, it may be premature to regard SLC3037 as a NEK7 inhibitor. If NEK7 inhibitors without effects on kinase activity could be developed, such compounds could be valuable candidates against gout or other diseases characterized by the inflammasome such as cardiovascular diseases, metabolic syndrome, and neurodegenerative disorders. In contrast to the results showing no role of kinase activity of NEK7 on the inflammasome, NEK7 phosphorylation has been reported to be important in the inflammasome (36). The detailed relationship between NEK7 kinase activity and phosphorylation and their roles in inflammasome remain to be clarified.

## Data availability statement

The original contributions presented in the study are included in the article/**Supplementary Materials**, further inquiries can be directed to the corresponding author/s.

## Ethics statement

Animal protocols were approved by the IACUCs of Hanyang University and the Department of Laboratory Animal Resources of Yonsei University College of Medicine. The study was conducted in accordance with the local legislation and institutional requirements.

## Author contributions

KP: Writing – original draft, Writing – review & editing. IS: Writing – original draft. YK: Writing – original draft. HK: Writing – original draft. SO: Writing – original draft. EJ: Writing – original draft. TS: Writing – original draft. JY: Writing – original draft. ML: Writing – original draft, Writing – review & editing.
