# Supplementary figures and images for: A novel NLRP3 inhibitor as a therapeutic agent against monosodium urate-induced gout
Source: Front Immunol. 2024 Feb 2;14:1307739. doi: 10.3389/fimmu.2023.1307739 (PMC10869544; doi:10.3389/fimmu.2023.1307739)

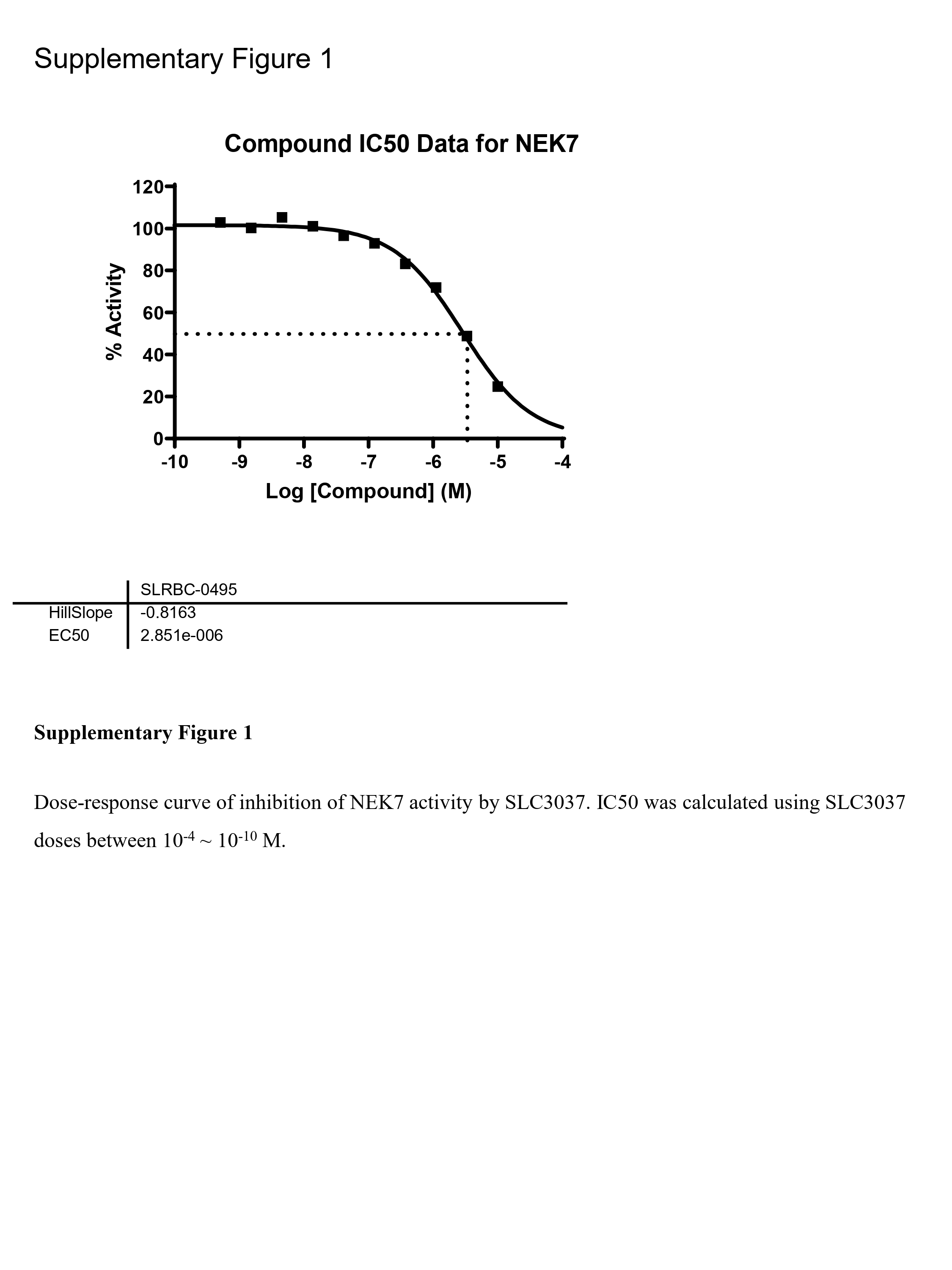

Supplement: Supplementary file 1 [file Image_1.jpeg]

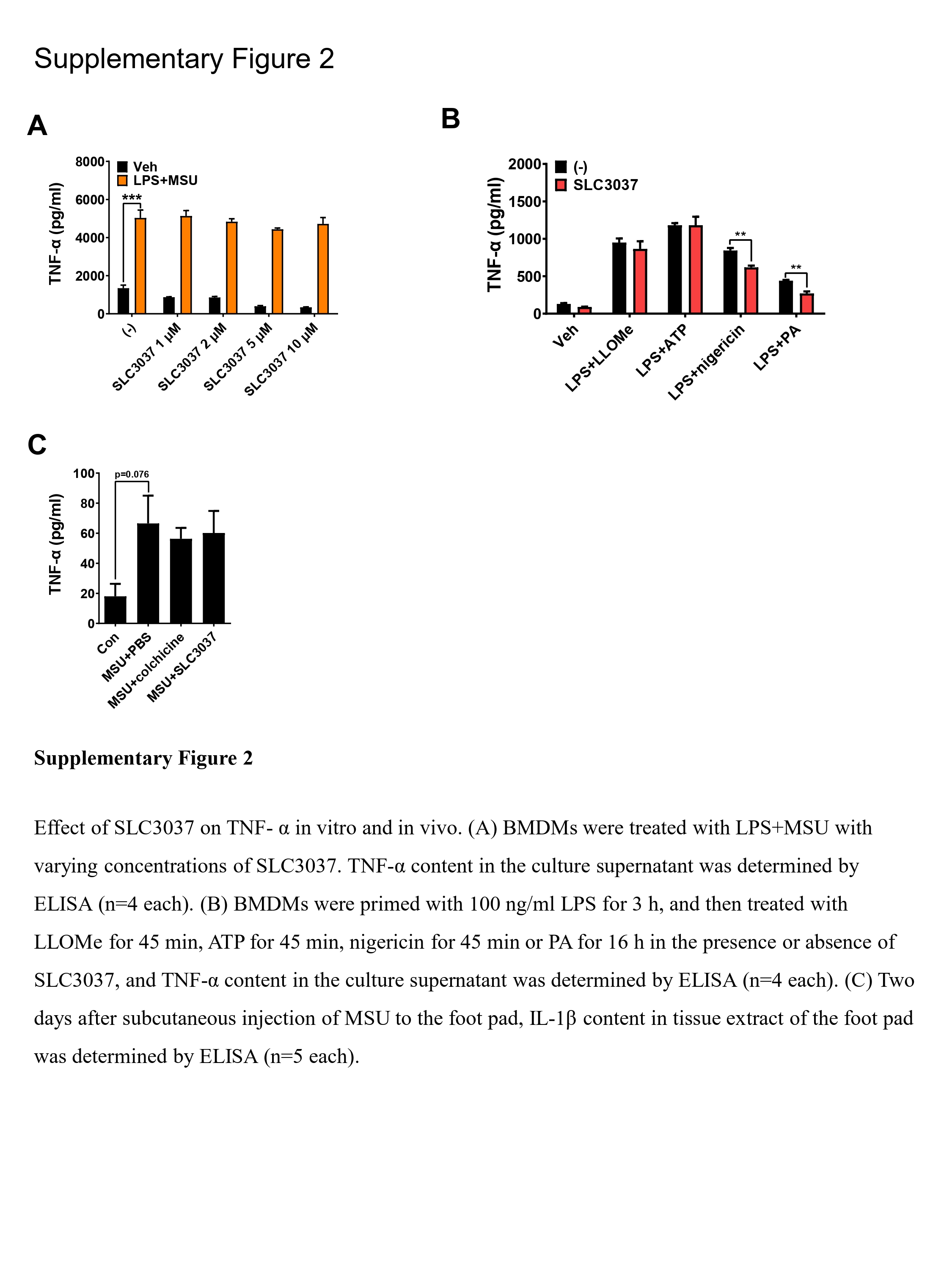

Supplement: Supplementary file 2 [file Image_2.jpeg]
